# The impact of COVID‐19 on a high‐volume incident learning system: A retrospective analysis

**DOI:** 10.1002/acm2.13653

**Published:** 2022-05-26

**Authors:** Dustin J. Jacqmin, Jennie S. M. Crosby

**Affiliations:** ^1^ Department of Human Oncology University of Wisconsin‐Madison Madison Wisconsin USA

**Keywords:** Incident learning, COVID‐19, Radiotherapy

## Abstract

**Purpose:**

The purpose of this work was to assess how the coronavirus disease 2019 (COVID‐19) pandemic impacted our incident learning system data and communicate the impact of a major exogenous event on radiation oncology clinical practice.

**Methods:**

Trends in our electronic incident reporting system were analyzed to ascertain the impact of the COVID‐19 pandemic, including any direct clinical changes. Incident reports submitted in the 18 months prior to the pandemic (September 14, 2018 to March 13, 2020) and reports submitted during the first 18 months of the pandemic (March 14, 2020 to September 13, 2021) were compared. The incident reports include several data elements that were evaluated for trends between the two time periods, and statistical analysis was performed to compare the proportions of reports.

**Results:**

In the 18 months prior to COVID‐19, 192 reports were submitted per 1000 planning tasks (*n* = 832 total). In the first 18 months of the pandemic, 147 reports per 1000 planning tasks were submitted (*n* = 601 total), a decrease of 23.4%. Statistical analysis revealed that there were no significant changes among the data elements between the pre‐ and during COVID‐19 time periods. An analysis of the free‐text narratives in the reports found that phrases related to pretreatment imaging were common before COVID‐19 but not during. Conversely, phrases related to intravenous contrast, consent for computed tomography, and adaptive radiotherapy became common during COVID‐19.

**Conclusions:**

The data elements captured by our incident learning system were stable after the onset of the COVID‐19 pandemic, with no statistically significant findings after correction for multiple comparisons. A trend toward fewer reports submitted for low‐risk issues was observed. The methods used in the work can be generalized to events with a large‐scale impact on the clinic or to monitor an incident learning system to drive future improvement activities.

## 1 INTRODUCTION

Coronavirus disease 2019 (COVID‐19) was first identified in December 2019. In March 2020, COVID‐19 was declared a pandemic by the World Health Organization and remains an ongoing pandemic. The arrival of the COVID‐19 pandemic had an immediate and profound impact on the field of radiation oncology. This impact was accompanied by a flourishing of literature describing the effects of the COVID‐19 pandemic on radiation oncology departments worldwide. The first such publications came out of early pandemic hotspots such as China,^1^, ^2^ Italy,^3^, ^4^ and the United States.^5^, ^6^, ^7^, ^8^, ^9^ These publications describe best practices for operating radiation oncology departments during a growing pandemic. As the pandemic continued, the focus of new publications shifted to issues related to clinical decision‐making during the pandemic. Numerous publications focused on how to adapt radiotherapy during the COVID‐19 pandemic for a wide variety of body sites, including brain,^10^ head‐and‐neck,^11^, ^12^ breast,^13^, ^14^ lung,^15^ prostate,^16^ and rectal cancer.^17^ Additional publications focused on the role of fractionation more generally^18^ as well as novel side effects.^19^, ^20^

As the pandemic stretched on, the radiation oncology community began to study larger scale implications of the COVID‐19 pandemic. Much has been written about the impact of the pandemic on medical education, including the recruitment,^21^ training,^22^, ^23^ and certification^24^ of medical students and residents. The impact of COVID‐19 on medical physics has been widely discussed, covering topics such as general clinical practice,^25^ staffing,^26^, ^27^, ^28^ the impact of infection control measures on treatment delivery,^29^, ^30^ and academic medicine.^31^ Viscariello et al.^32^ investigated the potential risks associated with pandemic‐related radiation oncology workflow adaptations. Czmielewski et al.^33^ investigated whether remote planning affected the frequency of errors. There have been two reports on the pandemic's impact on incident reporting, one from the Radiation Oncology Incident Learning System (RO‐ILS) database (5 months before and during COVID‐19)^34^ and another from a single institution's incident learning system (11 months before and during COVID‐19).^35^

The COVID‐19 pandemic caused many changes in our clinic, including variable patient volume, a shift toward hypofractionation, changes in on‐site staff availability, among many others. The purpose of this work is to assess how these changes impacted incident reporting and quality improvement activities that occur within our incident learning system. The operation of an electronic incident reporting system has allowed us to track the impact of pandemic‐driven changes on clinical operations and patient safety. This work reports the trends that have been observed in our incident reporting system prior to and during the COVID‐19 pandemic, as well as the error prevention interventions that have been implemented to address incident reports.

Ford and Evans^36^ provide an excellent review of incident learning systems in radiation oncology, including a comprehensive literature review and recommendations for implementing incident learning in the clinic. Their work cites over 30 articles describing institutional experience with incident learning systems. This work differs from these previous publications in two regards. First, this report describes how a high‐volume incident learning system responded to a major exogenous event such as the COVID‐19 pandemic. Second, our report characterizes the error prevention interventions that were implemented in response to our incident reports, allowing us to share how the effectiveness of the quality improvement team was impacted by COVID‐19.

## 2 METHODS

Our clinic began operating an electronic incident reporting system in January 2017. There have been over 2200 report submissions to date. Any staff member may voluntarily submit a form to report a deficiency in clinical operations, a patient safety issue, suggestion for improvement, or any information the staff member wishes to convey to our quality assurance (QA) committee. Our QA committee is composed of 14 members, including physicists, physicians, dosimetrists, nurses, scheduling/front desk staff, radiation therapists, department leadership, a physics resident, and a physician resident.

On the evening of March 12, 2020, the governor of Wisconsin issued Executive Order 72^37^, ^38^ proclaiming a public health emergency in response to COVID‐19 for the state where our clinic is located. We analyzed trends within our electronic incident reporting system to evaluate the impact of the COVID‐19 pandemic and its associated clinical changes. We divided reports into two groups: before COVID‐19 and during COVID‐19. The “before COVID‐19” group includes reports submitted in the 18 months prior to the executive order (September 14, 2018 to March 13, 2020), while the “during COVID‐19” group includes reports submitted during the subsequent 18 months (March 14, 2020 to September 13, 2021). Each report in this 3‐year interval was reviewed by at least one member of the QA committee and audited by at least one of the authors (both of whom are physicists) to ensure that the report data were complete and accurate. The authors worked closely to ensure that the same definitions were applied across all the reports. The following data elements were analyzed for trends: where in the radiation therapy workflow the event was discovered, where the event occurred, anatomical site, the role of the reporter, the event classification, whether a dosimetric change was required because of the issue, the patient safety risk, and the type of intervention implemented in direct response to the report.

Table 1 shows the list of data elements and their possible values. Most of the data element values are self‐explanatory. The possible values for most data elements (where discovered/occurred, role of reporter, event classification, dosimetric change, patient safety risk) are the same as those used in the RO‐ILS. The possible values for the type of intervention were derived from the Institute for Safe Medication Practices Medication Error Prevention “Toolbox.”^39^ The significance of the event in terms of risk to patient safety is classified according to the RO‐ILS response options of mild, moderate, or severe. In our clinic, we define risk in terms of the following question: “What would happen if the issue we discovered reached the patient and affected all fractions?” We refer to this as the potential severity of an event. We prefer potential severity to actual severity because using potential severity allows us to recognize the sources of high‐severity issues and implement preventative measures proactively. A potential severity of “mild” includes issues that did not have the potential to impact dosimetric or spatial accuracy of treatment or otherwise cause harm. A potential severity of “severe” includes events that have the potential to result in a dose deviation greater than 5% or a geometric miss of the target, either of which may affect the outcome of treatment. We also include acute, non‐radiotherapeutic hazards (magnetic resonance imaging safety, risk of falls, etc.) in the “severe” category. A potential severity of “moderate” includes events that fall between severe and mild.

**TABLE 1:** Data elements used in this study, including all possible parameter values

Classified by	Data element	Possible values
Report author	Where in the radiation therapy process the issue was discovered/occurred	Before simulation, preplanning imaging and simulation, treatment planning, pretreatment QA review, treatment delivery including imaging, on‐treatment QA, after the treatment course is finished, equipment and software QA, outside the radiation therapy workflow
	Anatomical site	Abdomen, brain, breast, extremities, gastrointestinal, genitourinary, gynecologic, head and neck, pelvis, skin, thorax, other, N/A
	Role of reporter	Administrator, dosimetrist, front desk staff, nurse, patient or patient representative, physician, physicist, radiation therapist, other
QA committee	Event classification	Therapeutic radiation incident, other safety incident, near‐miss, unsafe condition, operational/process improvement
	Dosimetric change required	Yes, no
	Patient safety risk	Mild, moderate, severe
	Type of intervention	None; education and information; rules and policies; independent double check systems; protocols, standards, and information; automation and computerization; forcing functions and constraints

Abbreviations: H&N, head‐and‐neck; QA, quality assurance.

The number of reports in each category was normalized to account for differences in clinical activity between the pre‐ and during COVID‐19 time intervals. The raw numbers of reports in each category were divided by the number of planning tasks completed during each interval, expressed in units of “reports per 1000 planning tasks.” In our clinic, a completed “planning task” is equivalent to a single traversal of the treatment planning workflow, which includes the following: computed tomography (CT) simulation in one position, at most one physics consult for image registration, one set of planning structures, one or more treatment plans, one physician plan review, one export of plans from the treatment planning system to the oncology information system, one physics plan review, and zero or more patient‐specific QA measurements. If a patient has multiple simulations, resulting in multiple sets of contours, there will be a separate planning task for each simulated position. Adaptive planning also generates a unique planning task. We address the advantages and disadvantages of normalizing by planning tasks in the discussion. The only category that was not normalized by planning tasks was the type of intervention implemented by the QA committee. This category was normalized by the total number of reports in each interval, expressed as a percentage of reports.

In addition to analyzing the categorical data as described above, we also analyzed the free‐text narratives in the reports. The most valuable part of a report is the narrative provided by the staff member. Narrative data are difficult to summarize and quantify, but we attempted to do so using the methodology described by Price et al.^40^ The narrative in each report was preprocessed by converting the text to lower case and removing punctuation. Next, common English language stop words (the, at, is, of, etc.) were removed. We also removed several radiotherapy words and abbreviations, such as therapist (and RTT), physician (and MD), physicist, etc., that were common in the narratives but did not add much context. From the remaining words, we generated two‐ and three‐word phrases and counted the frequency of each phrase. All text processing was performed in Python with Natural Language Toolkit (NLTK).^41^ Finally, the frequency distributions were converted into word clouds for visualization using WordCloud for Python.^42^ A minimum frequency of three was required to be included in the word cloud.

A series of two‐sample tests of proportions were performed to compare the rates of report submissions, adjusted for clinical load (per 1000 planning tasks), between the “before COVID‐19” and “during COVID‐19” groups for each of the 57 incident report categories (the “possible values” in Table 1). The resulting *p*‐values were adjusted to correct for multiple testing using the standard Benjamini Hochberg method for controlling the false discovery rate.^43^ All statistical tests were two sided, and a significance level of 5% (*p* < 0.05) was used for all tests. All analyses were conducted using R (version 4.1.1).^44^ The free‐text narrative data were not subjected to statistical analysis.

## 3 RESULTS

**FIGURE 1:**
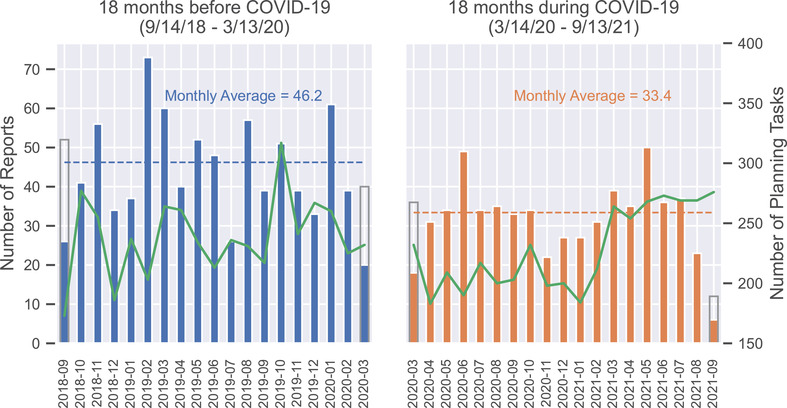
The number of incident reports submitted (blue/orange bars) and the number of planning tasks (green line) per month. The average number of incident reports submitted in the 18 months before coronavirus disease 2019 (COVID‐19) was 46.2 per month versus 33.4 per month in the 18 months during COVID‐19. The transition date between pre‐ and during COVID‐19 occurred in the middle of March 2020, resulting in half‐months of data at the edges of each subplot. The gray rectangles for these months represent double the number of reports during the half‐month, which gives a better sense of the trend over time

Figure 1 shows the number of reports per month during the time interval of the study. The overall number of reports decreased from 832 in the 18 months before COVID‐19 to 601 during COVID‐19, a decrease of 27.8%. The number of planning tasks before and during COVID‐19 was 4344 and 4080, respectively. The rate of reports per 1000 planning tasks was 192 versus 147, respectively, a decrease of 23.4%.

**FIGURE 2:**
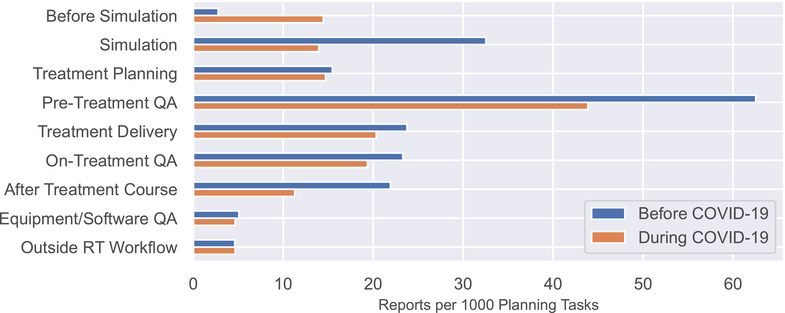
The number of reports submitted per 1000 planning tasks categorized by where in the radiotherapy process the issue was discovered. Although not statistically significant, we observed a large increase during coronavirus disease 2019 (COVID‐19) for events discovered before simulation and a large decrease in events discovered at simulation and during pretreatment quality assurance (QA)

Figure 2 shows the rate of report submission as a function of where in the radiotherapy process the issue was discovered. Discovery at the “before simulation” process step increased from 2.8 to 14.5 reports per 1000 planning tasks. This difference was statistically significant before correction (unadjusted *p*‐value = 0.002) but did not remain significant after application of the Benjamini Hochberg method (adjusted *p*‐value = 0.128). All other changes were insignificant.

**FIGURE 3:**
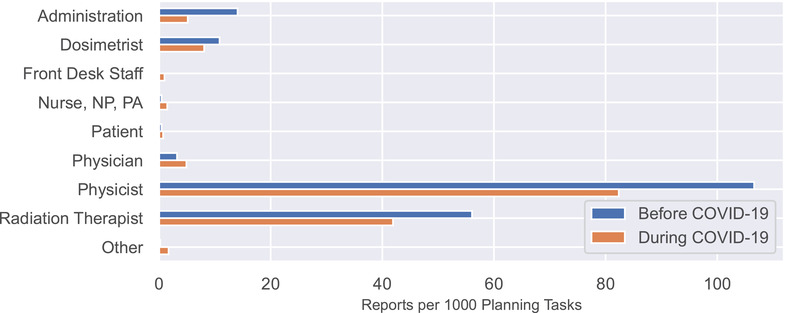
Reports per 1000 planning tasks categorized by the role of the reporter. NP, nurse practitioner; PA, physician assistant

Figure 3 shows the rate of report submission as a function of the role of reporter. Reports submitted by physicists decreased from 107 to 82 reports per 1000 planning tasks, the largest numerical decrease. Reports submitted by radiation therapists, the second largest source of reports, decreased as well (56 vs. 42). None of the differences was statistically significant.

**FIGURE 4:**
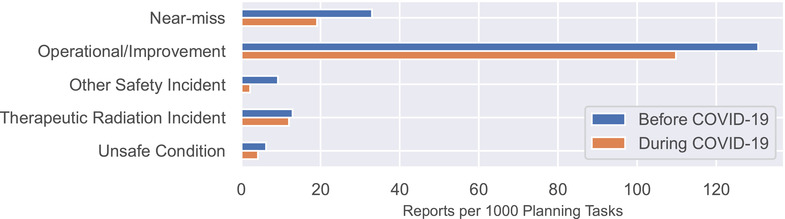
Reports per 1000 planning tasks categorized by event classification

Figure 4 shows the rate of report submission as a function of event classification. Reports classified as “operational/process improvement” decreased from 131 to 110 reports per 1000 planning tasks, the largest numerical decrease. Reports classified as “near‐miss” decreased from 33 to 19 reports per 1000 planning tasks, a decrease of 42%. Reports classified as “therapeutic radiation incident” were stable, with 13 reports per 1000 planning tasks before COVID‐19 and 12 reports per 1000 planning tasks during COVID‐19. None of the differences was statistically significant.

**FIGURE 5:**
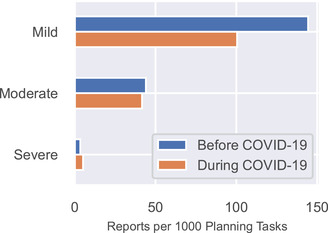
Reports per 1000 planning tasks categorized by their potential severity

Figure 5 shows the rate of report submission as a function of potential severity. Reports classified as “mild” decreased from 144 to 100 reports per 1000 planning tasks, the largest numerical decrease. Reports classified as “moderate” decreased slightly (44–42), and reports classified as “severe” increased slightly (3.5–5.2). None of the differences was statistically significant.

The number of reports that resulted in a dosimetric change to a patient's treatment plan decreased slightly, with 17 per 1000 planning tasks before COVID‐19 and 15 per 1000 planning tasks during COVID‐19. Reports that did not require a plan change dropped from 175 to 132 per 1000 planning tasks. These changes were not statistically significant. No statistically significant changes were seen with respect to where events occurred and anatomical site.

**FIGURE 6:**
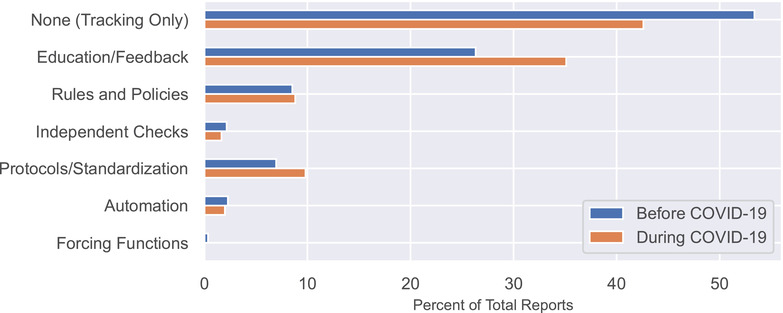
The types of interventions implemented in response to incident reports as a percentage of total reports

Figure 6 shows the types of intervention implemented in direct response to the report. The proportion of events addressed with a quality improvement intervention increased from 46.6% before COVID‐19 to 57.4% during COVID‐19. The largest increase was the use of “education and information” (26.3%–35.1%). This difference was statistically significant before correction (unadjusted *p*‐value = 0.044) but did not remain significant after application of the Benjamini Hochberg method (adjusted *p*‐value = 1.0). All other changes were insignificant.

**FIGURE 7:**
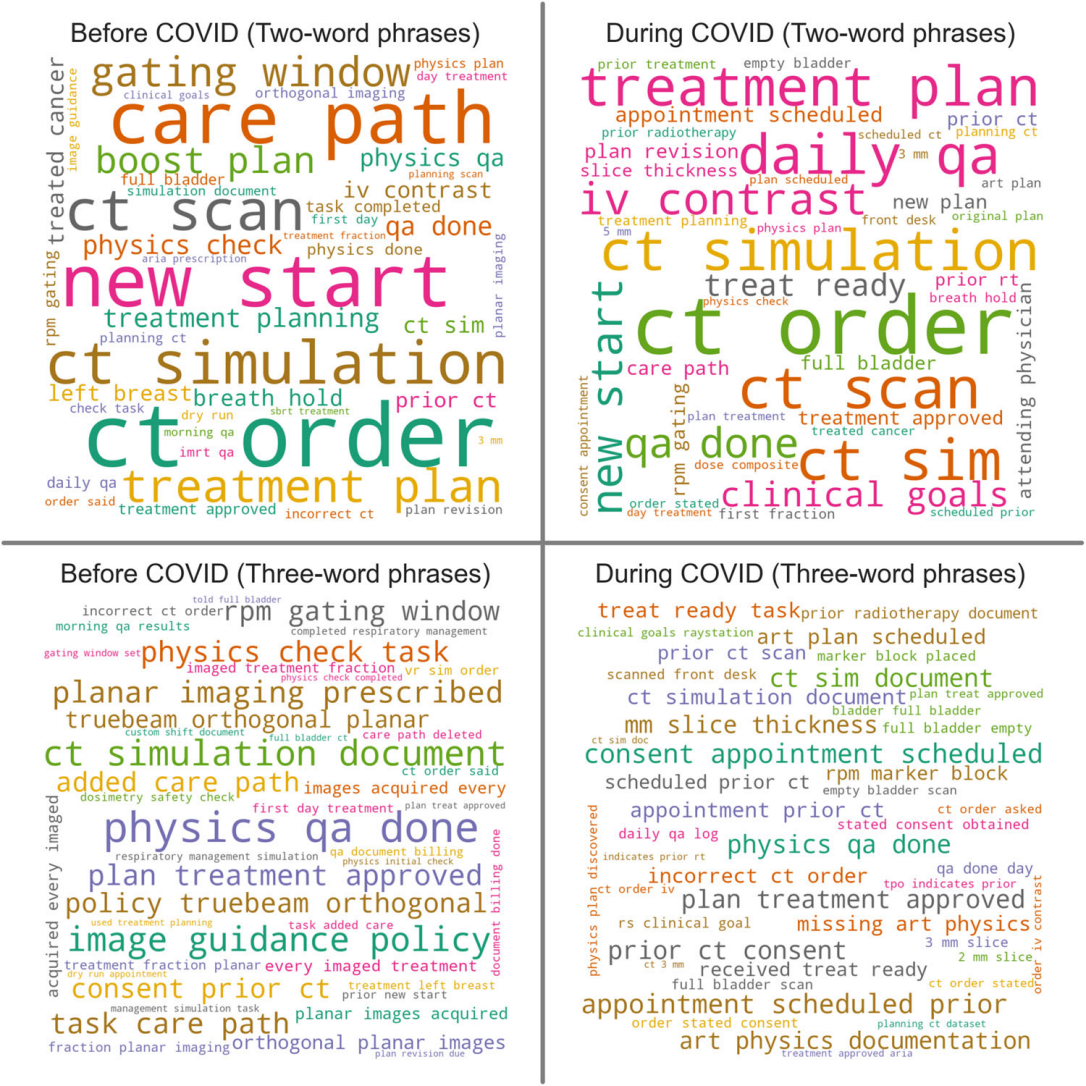
Word clouds showing the frequency of two‐ and three‐word phrases included in the free‐text narrative submitted with the incident report

Finally, Figure 7 shows word clouds composed of two‐ and three‐word phrases before and during COVID‐19. There were several common phrases related to CT simulation (“ct order,” “iv contrast,” “slice thickness,” “prior ct consent”), respiratory gating (“rpm gating,” “left breast,” “marker block placed,” “gating window”), pretreatment imaging (“orthogonal planar images,” “planar images acquired”), and adaptive radiotherapy (“art physics documentation,” “art plan scheduled”). Phrases related to CT orders and respiratory gating appear common before and during COVID‐19. Phrases related to pretreatment imaging are common before COVID‐19 but not during. Phrases related to intravenous (IV) contrast, consent for CT, and adaptive radiotherapy were not common before COVID‐19 but became common during.

## 4 DISCUSSION

The COVID‐19 pandemic has affected our clinic in many ways. We initially observed a decrease in patients under treatment, followed by an increase to the above historical levels that persisted for over a year. The distribution of staff on‐site has changed, with dosimetry and physics working off‐site on a regular basis. We have also seen the use of hypofractionation increase, particularly for breast cancer. We have transitioned to virtual group meetings, which work better for staff members who have a quiet space and a personal workstation to attend meetings. This has negatively affected the ability of radiation therapists to engage in group meetings. One of the largest changes we have seen in our clinic during COVID‐19 is changes in staffing. Some changes were planned and happened independent of the pandemic, but others were direct or indirect consequences of COVID‐19.

The goal of this work was to assess how these changes impacted incident reporting and quality improvement activities that occur within our incident learning system. The changes due to COVID‐19 may impact both the rate and nature of patient safety issues in our clinic, as well as the willingness or ability of staff to report these incidents. Our incident learning data measure the composite of these influences, so we cannot use our data to rigorously prove that COVID‐19 impacted the rate of issues or staff engagement independently. However, the evaluation of incident learning data is often the first step in discovering opportunities for improvement, and complementary lines of inquiry can help discern the underlying causes of trends in incident learning data. We believe there is great value in studying the performance of one's incident learning system over time. The methods described in this work can be applied to other scenarios with a potentially large impact. This includes events such as opening a new center, introducing new treatment delivery technologies, and recovering from a natural disaster.

As part of our analysis, we chose to normalize the data to account for differences in clinical activity between the pre‐ and during COVID‐19 time intervals. Our decision to use “planning tasks” was motivated by several advantages. First, in our clinic, the completion of a “planning task” is equivalent to a single traversal of the treatment planning workflow, so it represents a single unit of many types of clinical work, including simulation, contouring, image registration, physician and physics plan review, and plan preparation in the oncology information system. In other words, there is a strong correlation between the number of completed planning tasks and real‐world clinical work. Second, the total number of planning tasks could be reliably determined over the full 3‐year period using a single database query in our oncology information system. This was not possible for some competing metrics. For example, the total number of plans created was not practical to compute because the multiple systems holding this information could not be queried using standard features of the software. Third, we knew from our historical data that incident reports related to treatment planning were the most common type of report, so normalizing by a unit of treatment planning work was a way account for differences in clinical activities that have the largest impact on incident reporting. In the time interval between the initiation of our in‐house incident learning system and the beginning of our study, the “treatment planning” process step was the most common process step for occurrence, accounting for 30.6% of the total. Reports that occurred in “treatment planning” were the most common during the study as well (30.7% pre‐COVID, 29.1% during COVID). This agrees with findings in the literature. An early analysis of data from RO‐ILS found that events in the “treatment planning and pretreatment review/verification” workflow steps were most common, accounting for 33% of all events.^45^ Nyflot et al.^46^ also found that “treatment planning” was the most common process step for occurrence, accounting for 32.7% of events. Clark et al.^47^ found that over 50% of events occurred during the “treatment preparation” phase, which includes treatment planning and pretreatment preparation and review.

The use of planning tasks for normalization also has disadvantages. First, a single planning task may result in more than one plan, so a planning task does not accurately quantify the total number of plans generated. In our clinic, this is somewhat alleviated for intensity‐modulated radiotherapy and volumetric‐modulated arc therapy because our treatment planning system allows simultaneous optimization of multiple plans on a single image set, enabling our dosimetrists to complete multiple plans in parallel. However, 3D conformal plans must be created individually. One could resolve this bias using the total number of treatment plans created in each time interval for normalization. However, this would introduce its own bias because the number of treatment plans does not scale linearly with other types of clinical work in the treatment planning workflow. For example, a single set of targets and normal structures will be shared among all plans, so adding an additional plan does not increase the workload associated with contouring. Another disadvantage of using the number of planning tasks for normalization is that it does not account for treatment delivery. Incident reports that occurred at treatment delivery represent approximately 14% of reports in our study. To address this concern, we compared the change in the number planning tasks to the change in the number of treated fractions between the pre‐ and during COVID‐19 time periods. The number of planning tasks changed from 4334 to 4080, a drop of 5.9%, while the number of treated fractions changed from 46 765 to 43 797, a decrease of 6.3%. Given that the two metrics decreased by roughly the same proportion, we do not expect that normalizing by treated fractions would have impacted our results. Normalizing by the number of treated fractions also has the disadvantage of being uncorrelated with the amount of work done in the treatment planning process, the most common source of incident reports. A third disadvantage of using planning tasks for normalization is that the amount of real‐world clinical work per planning task can vary considerably from case to case (for example, whole‐brain radiotherapy vs. multitarget radiosurgery). The average complexity of treatment plans may have changed between the pre‐ and during COVID time intervals, such that the amount of clinical work per planning task changed over time. We did not investigate this, so it remains a potential cofounding variable. Altogether, we feel that normalizing by planning tasks offers the best mix of advantages and disadvantages compared to competing options.

Overall, there was a 27.8% decrease in incident reports during the COVID‐19 pandemic, similar to the decrease reported by Chera et al.^34^ (23% reduction in event reporting for states not in COVID hotspots and 33% reduction for COVID hotspot states identified by the Centers for Disease Control and Prevention). Amos et al.^35^ reported a 30% decrease in the number of incident reports. We noted one trend that manifested itself in several data elements. In the event classification data, we observed the largest decreases in “near‐miss” and “operational” reports, which by definition do not reach the patient. In the potential severity data, we observed a decrease in the rate of reports classified as “mild.” Finally, the rate of reports that did not result in a dosimetric change to the patient's care plan also decreased. Taken together, this suggests a trend toward fewer reports being submitted during COVID‐19 for low‐risk issues and stable reporting for issues of higher impact on patient care. Chera et al. reported a similar trend; practices rated a larger proportion of their events as high severity than pre‐COVID,^34^ indicating a reduction of lower severity event reporting. This could be attributable to staff effort being refocused toward addressing COVID‐19 clinical changes. The types of incident reports that became less common during COVID‐19 tended to be about issues that cause confusion, inefficiency, and delays. We shared this trend with our staff and reiterated that the reporting of low‐impact issues is valuable because it allows the QA committee to address quality of life issues for both patients and staff.

This work allowed us to assess the impact of remote work on incident reporting. During the pandemic, the physics team transitioned to roughly one‐third of staff working remotely, while the dosimetry team transitioned to roughly one‐half of the staff working remotely. The physicists experienced a 22.7% decrease in incident reporting (107–82 reports per 1000 planning tasks), while the dosimetrists experienced a 25.4% decrease (10.8–8.1). The therapists who remained fully on‐site during the pandemic showed a similar decrease of 25.2% (56–42). Given similar decreases among the three groups, it appears that the introduction of remote work did not have a direct impact on incident reporting. Czmielewski et al.^33^ investigated the impact of remote work by comparing the rate of incident reporting before and during COVID‐19 in the treatment planning process, where remote work was most widespread. They reported no significant increase in the frequency of treatment planning‐related incidents or in the severity of reported events when comparing the year before COVID‐19 to a year during COVID‐19.

Summarizing the narrative data in the form of word clouds was quite effective at capturing the main topics of discussion in our QA committee during the time interval of the study. In the year before COVID‐19, we introduced a new pretreatment imaging policy for breast radiotherapy. The growing pains associated with its implementation can be seen in the pre‐COVID word cloud. The issues were resolved quickly, and these phrases did not appear during COVID‐19. Before COVID‐19, we also noticed an increase in reports related to CT simulation. This prompted us to initiate a more detailed study of the problem, which largely occurred after COVID‐19 started. This explains the emergence of terms such as “IV contrast” and “consent”; our radiation therapists began providing more detailed narratives, allowing us to learn that simulations were being delayed due to missing IV contrast orders and signed consents. This is also related to another finding in our study, the increase in events discovered before simulation. Our radiation therapists began checking patient preparation for simulation at the beginning of the day before our patients arrived for their simulation. The trends that are visible in the word cloud highlight a limitation of this study: it is not possible to completely isolate the effects of COVID‐19 from other unrelated clinical changes. Our clinic regularly implements small‐scale quality improvement interventions such as new imaging policies and self‐studies of processes. These changes inevitably appear in incident reporting data and represent a confounding variable. Regardless, the insights gleaned from compiling the word clouds are valuable and offer many opportunities for future quality improvement efforts.

Noticeably absent from our word clouds is any reference to the pandemic. Indeed, the word “COVID” appeared only once in the 601 reports submitted after COVID‐19 began. The report, submitted by a radiation therapist, shared that a radiation oncologist was not available to supervise a challenging simulation because they were at home under quarantine due to close contact with a patient who had COVID‐19. Another report described a similar situation; a radiation oncologist under quarantine did not review a patient's pretreatment imaging prior to the final fraction of treatment. In response to these reports, a “doctor of the day” role was established to ensure coverage for physicians who could not be present in the clinic. Following this intervention, we have not seen any reports describing similar issues.

This is among the first studies to share the distribution of error prevention interventions that were implemented in response to our incident reports. During the period of study, our clinic implemented an average of 244 interventions per year. The interventions range from simple and inexpensive (education, reminders, new rules) to complex and expensive (automation, forcing functions). Our use of interventions favors those that are easiest to implement, although we often escalate to more involved interventions when an issue persists. During COVID‐19, our data showed a 9% increase in the use of education and information. This generally took the form of continuing education and reminders at group meetings. In the early part of the pandemic, when our patient census was lower, our care team had more time for continuing education activities, which allowed us to significantly increase the use of this intervention.

## 5 CONCLUSION

This work showed that our incident reporting and quality improvement activities were stable during COVID‐19. There were no statistically significant changes in patterns of incident reporting and quality improvement activities after correction for multiple comparisons, suggesting that our clinic absorbed the stress of COVID‐19 very well. That said, not all deficiencies in clinical operations, patient safety issues and suggestions for improvement are submitted to our incident learning system, so the quantitative results must be considered with this caveat in mind. There were several qualitative trends that were of interest. We observed an overall decrease in the rate of incident reports, similar to trends reported by Chera et al.^34^ and Amos et al.^35^ There was a general trend toward fewer reports related to low‐risk issues. While not statistically significant, we found the trend compelling enough to address with our staff. The rate of incident reporting by therapists, physicists, and dosimetrists decreased by similar amounts during COVID‐19, despite differing amounts of remote work among the groups. The use of word clouds allowed us to summarize narrative data and view trending topics before and during COVID‐19. The study has made us more aware of our reliance on simpler error prevention tools, both before and during COVID‐19. We believe the methods used in this work may be generalized to events with a large‐scale impact on clinical operations. In the absence of such events, these methods may also be used to monitor one's incident learning system and drive future continuous improvement activities.

## AUTHOR CONTRIBUTIONS

Dustin J. Jacqmin made substantial contributions to the conception and design of the research project. This included the acquisition, analysis, and interpretation of data for the manuscript. He was involved in drafting the work and revising it critically for important intellectual content. He gave final approval of the version to be published and agrees to be accountable for all aspects of the work in ensuring that questions related to the accuracy or integrity of any part of the work are appropriately investigated and resolved. Jennie S.M. Crosby made substantial contributions to the conception and design of the research project. This included the acquisition, analysis, and interpretation of data for the manuscript. She was involved in drafting the work and revising it critically for important intellectual content. She gave final approval of the version to be published and agrees to be accountable for all aspects of the work in ensuring that questions related to the accuracy or integrity of any part of the work are appropriately investigated and resolved.

## CONFLICT OF INTEREST

The authors have no conflicts of interest to disclose.
